# Graphene-Modified Titanium Surface Enhances Local Growth Factor Adsorption and Promotes Osteogenic Differentiation of Bone Marrow Stromal Cells

**DOI:** 10.3389/fbioe.2020.621788

**Published:** 2021-01-12

**Authors:** Jiayu Lu, Jiayue Sun, Derong Zou, Jinlin Song, Sheng Yang

**Affiliations:** ^1^Department of Stomatology, Shanghai Jiao Tong University Affiliated Sixth People's Hospital, Shanghai, China; ^2^College of Stomatology, Chongqing Medical University, Chongqing, China; ^3^Chongqing Key Laboratory of Oral Diseases and Biomedical Sciences, Chongqing Medical University, Chongqing, China; ^4^Chongqing Municipal Key Laboratory of Oral Biomedical Engineering of Higher Education, Chongqing Medical University, Chongqing, China

**Keywords:** graphene, graphene coating, concentrated growth factors, osteogenic differentiation, bone marrow stromal cells

## Abstract

Graphene coating exhibits excellent abilities of protein adsorption and cell adhesion, which might expand the osteogenic activity of titanium implant surface to adapt to the environment of low bone mass and poor bone quality. In this paper, we designed and explored the graphene-coated titanium sheet, through the surface modification of oxygen-containing functional groups, to optimize the adsorption capacity of material by improving the electrostatic interactions, and successfully adsorbed and sustained-released a variety of osteogenic related growth factors in the autologous concentrated growth factors. Compared with the pure titanium, we observed that the bone marrow stromal cells (BMSCs) on the graphene-coated titanium with concentrated growth factors showed a flat shape and expressed osteogenic related genes and proteins, while the coating surfaces promoted and accelerated the osteogenic differentiation ability of BMSCs. The results suggested that it might be a feasible alternative to improve the osteogenesis of dental implant in the early stage.

## Introduction

With the rapid development of surface nano-modification technology, metal implants have become the main orthopedic biomaterials (Chrcanovic et al., 2014). SLA (**S**andblasting, **L**arge grid, **A**cid-etched) treated surface metal implants prepared by acid etching and roughening can increase the implant-bone interface contact area by more than 60% due to the preparation of a porous titanium implant surface topography, and significantly enhance the torque resistance of the implant and greatly increase the success rate (Li et al., 2002). However, improving the osseointegration ability of metal materials, enhancing local cytokine enrichment, and improving the osteogenic microenvironment around the implant are very important to further improve the osteogenesis ability of the implants.

Growth factors or cytokines can be used as biological signal initiating factors in a specific microenvironment to promote the mobilization of endogenous stem cells in the body, regulate tissue cells to quickly adapt to implants, and promote osseointegration and bone defect repair (Varkey et al., 2004; Tayalia and Mooney, 2009). The use of safe and reliable concentrated growth factors excludes the possibility of immune rejection and cross-infection with exogenous growth factors (Chen et al., 2010). Due to the lack of anticoagulants, the coherent fibrin scaffolds in current platelet-rich products including platelet-rich fibrin (PRF), advanced platelet-rich fibrin (A-PRF), and concentrated growth factors (CGF) prevent binding to the bone grafts. Liquid concentrated growth factor preparations such as injectable PRF (i-PRF) and liquid CGF, the sparse fibrin networks of which contain high concentrations of multiple growth factors, can be used alone or as a supplement to other biomaterials in regenerative medicine to promote tissue regeneration (Simonpieri et al., 2012; Mourao et al., 2015; Murray, 2018).

Building materials with adsorption capacity on the surface of the implant is an effective way to enrich the above-mentioned various cytokines and proteins. Graphene coating has become an excellent candidate material for its high strength, large specific surface area, and porosity (Yan et al., 2017; Stephens-Fripp et al., 2018; Wu et al., 2019; Xing et al., 2019). It has also generated great expectations in the biomedical field due to its extraordinary mechanical properties, biocompatibility, electrical conductivity and so on (Liao et al., 2018; Ryan et al., 2018). In particular, graphene has broad application prospects in the field of biomaterials. Graphene is reported to have osteoinductivity, which promotes osteogenesis and osseointegration and further repairs bone defects (Lu et al., 2016; Holt et al., 2017). The cells on the surface exhibit enhanced proteins adsorption and osteogenic activity owing to the large surface area and oxygen-containing functional groups of graphene (Dubey et al., 2015).

Our research uses graphene coating technology to increase the electrostatic and chemical functional groups on the surface of the titanium plate to optimize the adsorption capacity. After interacting with autologous concentrated growth factors, the graphene-coated titanium can successfully adsorb and sustained release several osteogenic related growth factors. The results revealed that BMSCs on the graphene- coated titanium sheets exhibited flattened shapes and expressed more obvious osteogenic related genes and proteins compared to the pure titanium. The RhoA/ROCK1/ERK1/2 signaling pathway related to cytoskeleton deformation was also up-regulated. In addition, the adsorption of various growth factors on the surface of graphene-coated titanium sheet could further promote and accelerate the osteogenic differentiation of BMSCs. The results suggested that the graphene-coated titanium with concentrated growth factors may enhance the osteogenic ability as bone tissue engineering scaffold, bone repair, dental implant and bone graft materials.

## Materials and Methods

### Preparation of Graphene-Coated Titanium Sheets

The surface of titanium sheet was treated by SLA, and immersed in 3% ethanol solution of 3-aminopropyltriethoxysilane (3-APTES, 3% ethanol solution of APTES) for 30 min to improve the adhesion between graphene and titanium. Then reduced graphene oxide (rGO) nanosheets were loaded on the titanium sheets by immersing the functionalized samples into the rGO solution (0.4, 0.04, and 0.004 mg/ml) for 1 h, which were synthesized by chemical reduction of a GO solution acquired by chemical oxidation and exfoliation of natural graphite using our previously reporte d method and characterized by atomic force microscopy (AFM) (Lu et al., 2016). The synthesized graphene-coated titanium sheets were then cleaned under ultrasonic for 30 min to remove the uncoated rGO nanosheets.

### Characterization of Graphene-Coated Titanium Sheets

Raman spectroscopy (Bruker Optic SENTERRA, R-200L) was used to identify the surface characteristics of graphene-coated titanium sheets at room temperature with a laser wavelength of 633 nm. The chemical compositions of the samples were analyzed by X-ray photoelectron spectroscopy (XPS, AXIS Ultra DLD, Kratos). Fourier transform infrared radiation (FTIR) was conducted by a Perkin Elmer Spectrum 100 analyzer. The conductivity of the samples was measured by using a four-probe technique (Jandel Model RM3000). A field emission scanning electron microscope (SEM, Nova NanoSEM, NPE218) was used to observe the morphology of the samples.

### Biocompatibility of Graphene-Coated Titanium Sheets

In order to isolate bone marrow stromal cells (BMSCs), 5 ml bone marrow was collected from rabbit iliac crest of 12-month-old rabbits (average weight 2.5 kg) and cultured in Dulbecco modified Eagle medium (DMEM) (Gibco, USA) containing 10% FBS (Gibco, USA) in 5% CO_2_ incubator. The medium was changed every 2 days. When the cells reached 80% confluence, the cells are passaged. Animal experiments were approved by the Animal Welfare Ethics Committee of Shanghai Sixth People's Hospital affiliated to Shanghai Jiao Tong University, School of Medicine (No: DWLL2020-0577).

All samples were sterilized by autoclaving (125°C/0.14 MPa, 30 min) before use. The BMSCs were cultured on the graphene-coated titanium sheets for 2 days. The potential cytotoxic effects of the samples were assessed using the Live/Dead Staining Kit (ScienCell, USA) according to the manufacturer's instructions. Live cells were stained with the fluoresces green polyanionic dye calcein, while dead cells with damaged membranes allowed EthDIII to enter and bind to nucleic acids fluoresced red under the fluorescence microscope (DMI6000B; Leica, Germany).

### Adsorption and Sustained Release of Concentrated Growth Factors by Graphene-Coated Titanium Sheets

After intravenous anesthesia with pentobarbital sodium (30 mg/kg), chest hair of 12-month-old rabbits (average weight 2.5 kg) was taken and skin was disinfected. Then, 10 ml of whole blood was extracted from the heart and transferred to a centrifuge tube containing an anticoagulant (heparin lithium). At centrifuging at 3,000 rpm for 10 min (TR-18, Trausiam, China), 1 mL of liquid containing concentrated growth factors was collected from 3 mm above the red blood cell aggregation on the bottom. 800,000U of penicillin was injected to prevent infection for 3 days.

The graphene-coated titanium sheets were immersed in the concentrated growth factors at 37°C for 2 days for adsorption. Then the remaining liquid was collected. The growth factors of original and remaining concentrated growth factors including PDGF, VEGF, TGF-β, IGF, FGF, and BMP-2 were quantified by ELISA assay according to the manufacturer's protocol. In short, 40 μL of test dilutions and 10 μL of the sample were co-cultured in 96 well antibody precoated plates at 37°C for 30 min. Wells then were washed five times. After incubation with enzyme solution in the dark for 15 min, 50 μL stop solution was added to stop the enzyme reaction. The absorbance was measured at 450 nm with a microplate reader (iMark, Bio-Rad, USA). All samples were measured in triplicate.

The graphene-coated titanium sheets with concentrated growth factors were placed in 2 mL PBS to allow the growth factors to release. At each time point (1, 2, 7, and 14 days), the supernatant was collected. Sustained release of PDGF, VEGF, TGF-β, IGF, FGF, and BMP-2 were quantified by ELISA assay according to the manufacturer's protocol above.

### Morphological Changes of the BMSCs on Graphene-Coated Titanium Sheets With Concentrated Growth Factors

The BMSCs were cultured on samples with concentrated growth factors (2 days' adsorption) for 3 days, then fixed overnight with 2.5% glutaraldehyde at 4°C and freeze-dried (Alpha 1-2; Christ, Germany). Finally, the adhesion and growth of the BMSCs on the sheets were observed by field emission scanning electron microscope (SEM, Nova NanoSEM, NPE218).

The morphology of the BMSCs was observed by labeling the actin cytoskeleton with Phalloidia-TRITC (Sigma, USA). In short, the BMSCs were cultured on the samples with concentrated growth factors for 24 h, and then fixed in 4% paraformaldehyde at room temperature for 30 min. After 0.1% Triton X-100 for 20 min, the actin cytoskeleton was labeled by incubating with Phalloidia-iFluor 488 (Abcam, USA) for 30 min and visualized with a fluorescence microscope (DMI6000B; Leica, Germany).

### Osteogenic Differentiation of the BMSCs on Graphene-Coated Titanium Sheets

The BMSCs were seeded on the samples with concentrated growth factors and culture dishes in a growth medium or osteogenic medium (growth medium containing 50 μg/mL L-ascorbic acid, 10 mM glycerophosphate and 100 nM dexamethasone) (Su et al., 2020). ALP activity was evaluated as previously described on days 1, 4, and 7 after cell seeding. Briefly, the cells were isolated from the plate with trypsin/EDTA and resuspended in 0.2% NP-40 lysis buffer. Each sample was mixed with 1 mg/mL p-nitrophenyl phosphate (pNPP, sigma, USA) in 1 M diethanolamine as a substrate and incubated at 37°C for 15 min. The reaction was stopped by adding 3N NaOH. ALP activity was quantified by absorbance at 405 nm (ELX808; Bio-tek, USA). The total protein content of the same sample was determined by Bradford method with Bio-Rad protein analysis kit (Bio-Rad, USA), read at 630 nm and calculated according to a series of BSA (Sigma, USA) standards. ALP activity was calculated as the absorbance of 405 nm (OD value) per mg of total cell protein. All experiments were conducted in three times.

The BMSCs were seeded on the samples with concentrated growth factors and culture dishes in a growth medium or osteogenic medium for 21 days. The mineral deposits in the extracellular matrix were analyzed by staining with 10 mg/L Alizarin Red S for 5–7 days, and then visualized using a fluorescence microscopy (DMI6000 B, Leica, Germany).

The BMSCs were seeded on the samples with concentrated growth factors and culture dishes in a growth medium or osteogenic medium for 15 days. The cells on substrates were fixed by 4% paraformaldehyde for 30 min. The anti-BMP-2 and anti-RUNX-2 primary antibody (Abcam, USA) was added onto the samples at 4°C overnight. After rinsing, fluorescein isothiocyanate (FITC) goat anti-mouse second antibody (Abcam, USA) was added onto each chip and incubated at room temperature for 1 h. The chips were inverted onto glass slides mounted with Fluoroshield with DAPI (Sigma, USA) and visualized under a fluorescence microscope (DMI6000B, Leica, Germany).

The BMSCs were seeded on the samples with concentrated growth factors and culture dishes in a growth medium or osteogenic medium for 1, 7, and 15 days. Whole RNA was extracted from the cells according to the vendor's protocol (Invitrogen, USA). PrimeScript reagent kit of reverse reaction (Thermo Fisher Scientific, USA) was used to carry out reverse transcription reaction on RNA following the protocols of the manufacturer. Quantitative analysis on the change of expression level of ALP, BMP-2, ERK1/2, OCN, OPN, RhoA, ROCK, RUNX-2 genes (Table 1) was conducted by Maxima SYBR Green Master Mix (Thermo Fisher Scientific, USA) in ABI PRISM 9700 PCR (ABI, USA). Realtime RCR reaction was performed at 94°C for 10 min and 40 cycles of amplification, which consisted of denaturation step at 94°C for 15 s, annealing step at 65°C for 30 s, and extension step at 72°C for 30 s. The primer sequences were shown subsequently in Table 1. The relative expressions of these genes were normalized to the β-Actin gene expression. The change in the expression of mRNA was assessed by the 2-ΔΔCt approach.

**Table 1:** Primer sequences of osteogenic genes expressed by BMSCs.

**Gene Target**	**Primers sequence (5^**′**^-3^**′**^)**
ALP	Forward primer: 5′ CGTGGCAACTCCATCTT 3′ Reverse primer: 5′ AGGGTTTCTTGTCCGTGT 3′
BMP-2	Forward primer: 5′ TGAGGATTAGCAGGTCTTT 3′ Reverse primer: 5′ TGGATTTGAGGCGTTT 3′
ERK1/2	Forward primer: 5′ GCGTGGTGTTCAAGGT 3′ Reverse primer: 5′ TCTCGCCATCGCTGTA 3′
OCN	Forward primer: 5′ ACTCTTGTCGCCCTGCTG 3′ Reverse primer: 5′ TCGCTGCCCTCCCTCT 3′
OPN	Forward primer: 5′ TACCTTCTGATTGGGACA 3′ Reverse primer: 5′ CGAAATTCACGGCTCT 3′
RohA	Forward primer: 5′ CAAGATGAAGCAGGAGC 3′ Reverse primer: 5′ ACAAGACAAGGCACCC3 3′
ROCK	Forward primer: 5′ GTGAAGCCTGACAACA 3′ Reverse primer: 5′ CTCGTCCATAATAACCAT 3′
RUNX-2	Forward primer: 5′ GACCACCCAGCCGAACT 3′ Reverse primer: 5′ CAGCACCGAGCACAGGA 3′

### Statistical Analysis

All sample values were expressed as the Mean values ± standard deviation and analyzed by Prism software (GraphPad Prism 8). Statistically significant values were defined as *p < 0.05, ^**^p < 0.01, ^***^p < 0.001, and ^****^p < 0.0001 based on one-way analysis of variance (ANOVA).

## Results

### Preparation and Characterization of Graphene-Coated Titanium Sheets

**Figure 1:**
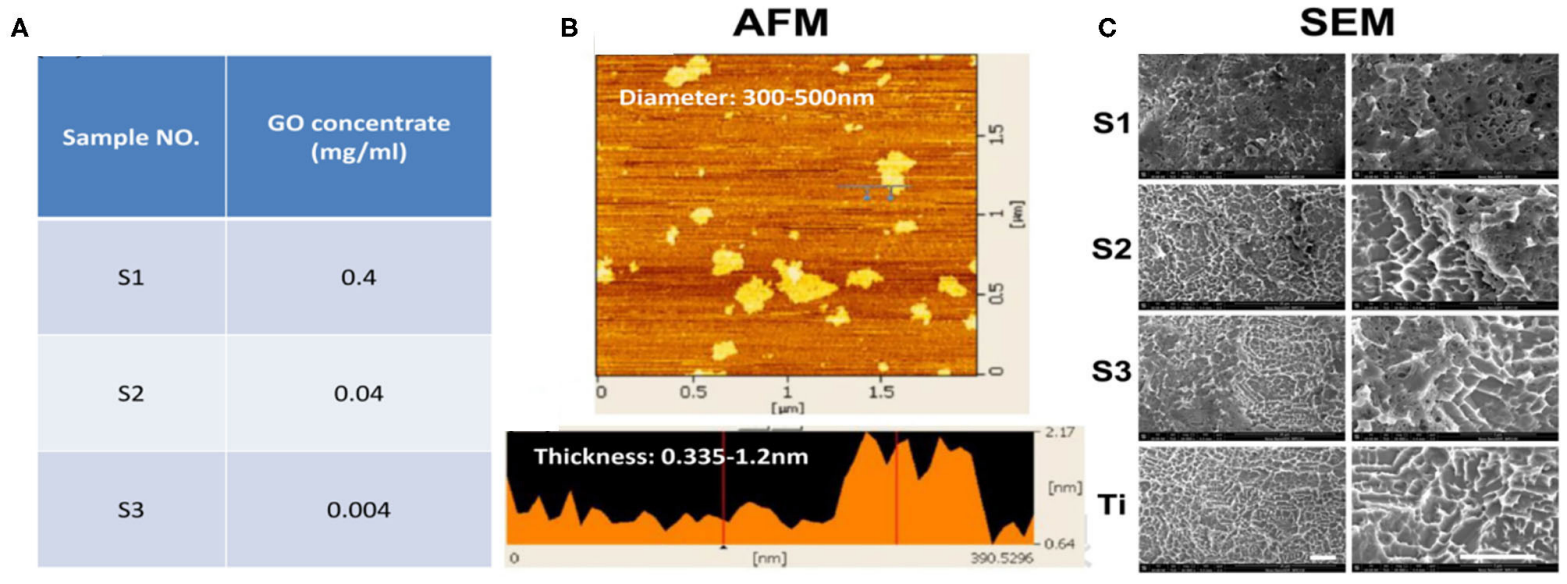
Preparation and SEM images of the graphene-coated titanium sheets. **(A)** Sample No. of **S1**, **S2**, and **S3** indicates the 0.4, 0.04, and 0.004 mg/ml of the rGO solution. **(B)** The thickness and the diameter of the loaded rGO nanosheets were characterized by AFM. **(C)** SEM images of graphene coating on SLA treated titanium sheets and pure SLA treated titanium (**Ti**). Scale bars: 5 μm.

The thickness of the loaded rGO nanosheets (0.4, 0.04, and 0.004 mg/ml) was about 0.335–1.2 nm and the diameter was about 300–500 nm (Figures 1A,B). SEM images (Figure 1C) clearly showed that the surface of titanium treated by SLA formed a porous structural morphology with a diameter of about 2 μm. It is reported that the biological activity of the implant surface and the bond strength of the implant-bone interface can be improved by roughening the surface. SEM also showed the graphene-coated titanium sheets with graphene coating attached on the porous surface (Figure 1C). On the S1, rGO coated the entire surface and stacked up. As the concentration decreased, the graphene distributed more evenly. On the S3, graphene concentration was too low and the surface of titanium exposed.

**Figure 2:**
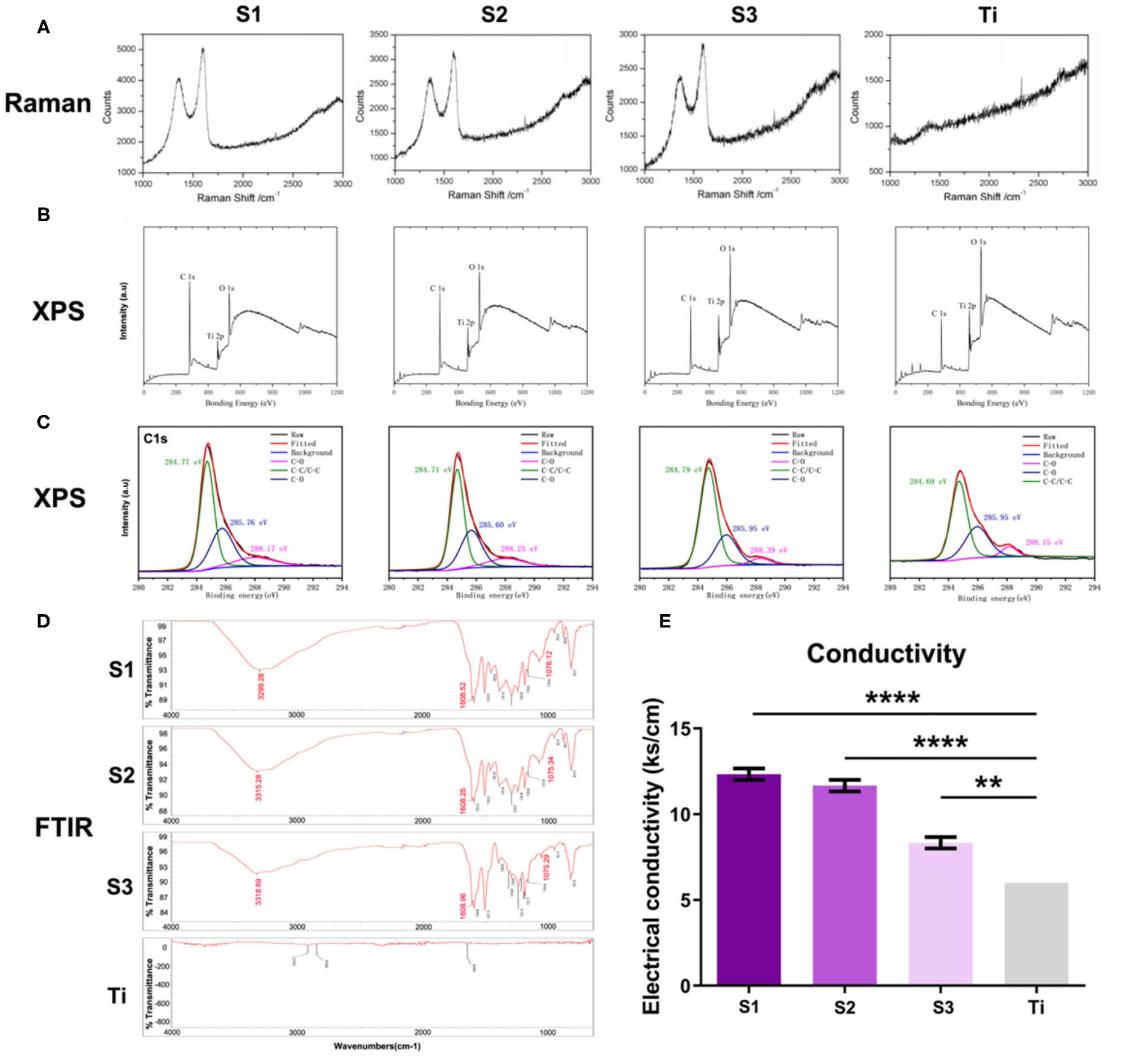
The characterization of the graphene-coated titanium sheets. **(A)** The Raman spectra, **(B)** XPS measurement spectra, **(C)** the high resolution XPS C1s core level spectra, **(D)** FTIR spectra and **(E)** the conductivity of graphene-coated titanium sheets (**S1, S2, and S3**) and titanium sheet (**Ti**). (**p < 0.01 and ****p < 0.0001).

The Raman spectra (Figure 2A) of S1–S3 samples showed the characteristic peaks of graphene at about 1,580 and 1,350 cm-1, revealing that their surfaces are all coated with graphene, while these two peaks are not seen on the surface of titanium sheet. As shown in Figure 2B, XPS measurement spectra of graphene-coated titanium sheets showed that the relative intensity of C1s/O1s peak decreased significantly with the decrease of loading concentration, indicating the weakening of oxygen functional groups. The high resolution XPS C1s core level spectra (Figure 2C) showed three peaks with centers at 284.7, 285.7 and 288.2 eV, corresponding to C-C/C=C, C-O and C=O (carbon-carbon bond, hydroxyl group and carbonyl group, respectively). With the decrease of the load concentration, the three peaks gradually weaken. FTIR spectra (Figure 2D) showed the characteristic peaks of rGO (C-OH at 3,315 cm-1, C=O of 1,608 cm-1 and epoxy resin of 1,075 cm-1), which means that functional rGO nanosheets have been successfully introduced into the surface of porous titanium sheets. With the increase of loading concentration, the conductivity of the sheets also increased (Figure 2E), which indicated that rGO nanosheets has been successfully introduced into the surface of porous titanium sheets and increased the conductivity of the sheets.

### Biocompatibility of Graphene-Coated Titanium Sheets

**Figure 3:**
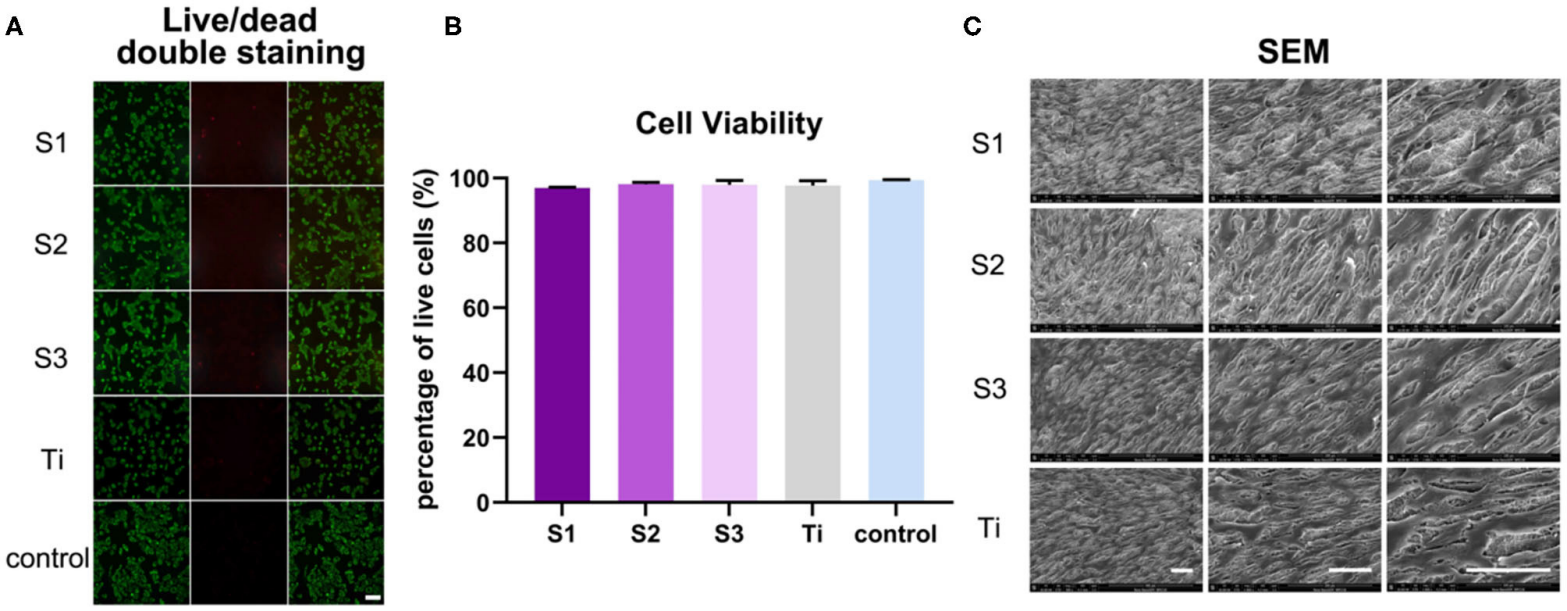
BMSCs viability and growth on graphene-coated titanium sheets and titanium sheet. **(A)** Live/dead double staining of BMSCs on different samples (live cells were stained florescent green, and dead cells appeared red). **(B)** Cell viability of BMSCs on graphene-coated titanium sheets and titanium sheet. **(C)** SEM images of adhesion of BMSCs onto graphene-coated titanium sheets and titanium sheet at 3 days after seeding. Scale bars: 100 μm.

After growing 2 days on the graphene-coated titanium sheets, the BMSCs in all groups showed green living cells, with only a few red dead cells, revealing that the graphene-coated titanium sheets had good biocompatibility and were suitable as implants for bone regeneration (Figure 3A). Cell viability, expressed as the ratio of live to dead cells, was 96.98 ± 0.22%, 98.09 ± 0.61%, 97.97 ± 1.35%, 97.72 ± 1.52%, and 99.40 ± 0.17% on samples S1–S3, titanium sheet and cell slide, respectively (Figure 3B), further confirmed the non-toxic to cells. After growing 3 days on the samples, SEM observation (Figure 3C) showed that the cells on each sample were spread out and appeared confluent, indicating that the cells grew well on the materials. Changes in cell morphology may be the result of a combination of rGO coating surface morphology, mechanical properties, and electrical conductivity. In general, the adhesion, adaptation and proliferation of BMSCs on the surface of graphene-coated titanium plates were similar to that of the titanium plate, which indicated that the graphene-coated titanium plates had good biocompatibility.

### Adsorption and Sustained Release of Osteogenic Growth Factors

**Figure 4:**
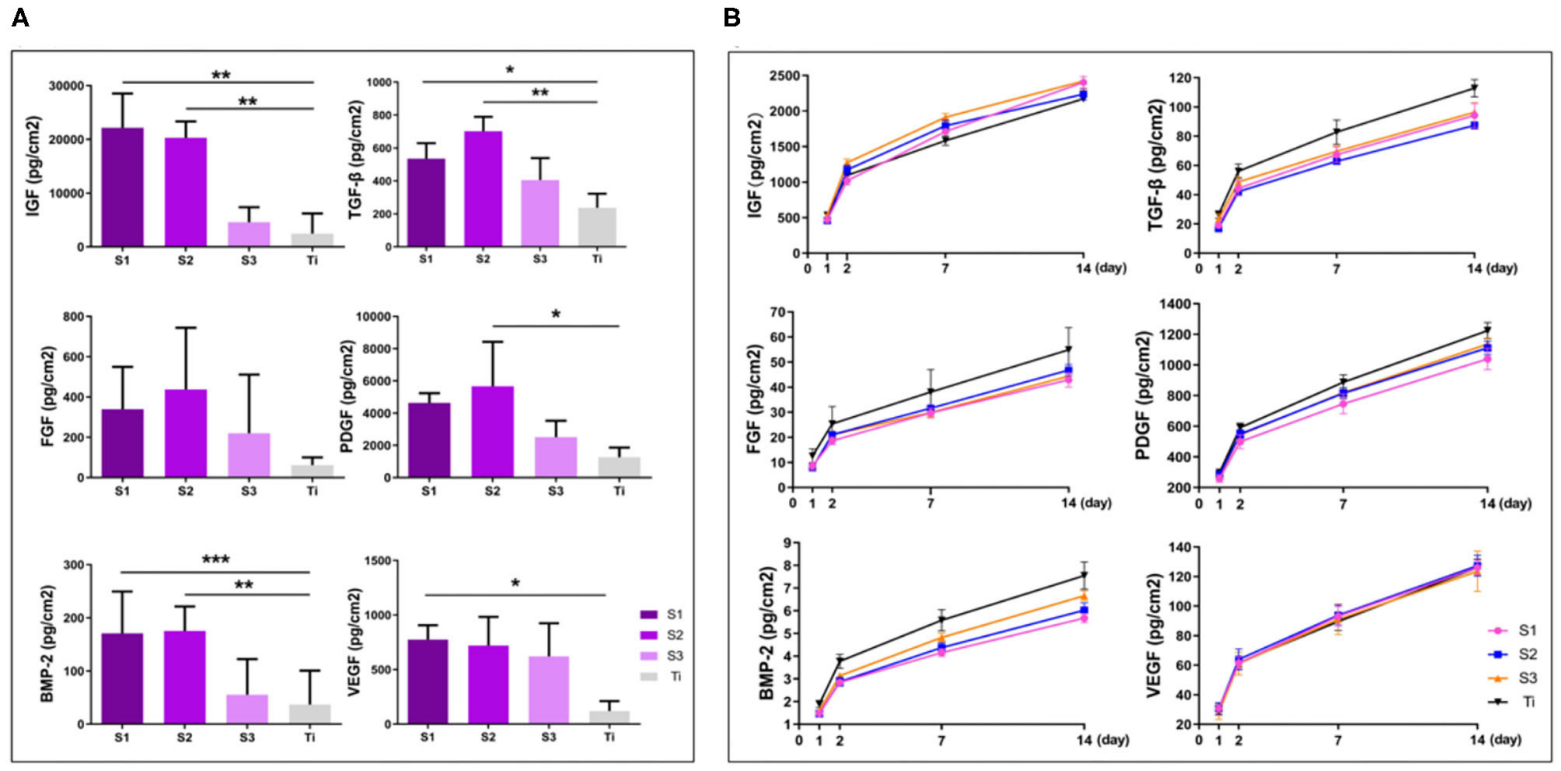
Adsorption **(A)** and cumulative sustained release **(B)** of osteogenic growth factors of graphene-coated titanium sheets and titanium sheet (*p < 0.05, **p < 0.01, and ***p < 0.001).

After centrifugation, concentrated growth factors can be easily obtained on the erythrocyte deposition layer. Immersed in the concentrated growth factors for 2 days for adsorption, the graphene-coated titanium sheets can adsorb more growth factors than the titanium sheet, especially TGF-β, IGF and BMP-2 (Figure 4A). We calculated the cumulative release of growth factors at day 1, 2, 7, and 14. The results showed that the sustained release of PDGF, VEGF, TGF-β, IGF, FGF, and BMP-2 in all groups over the 14-day observation period (Figure 4B). Since graphene absorbs growth factors through chemisorption such as electrostatic force and hydrogen bond. While the titanium plates lack these adsorption mechanisms and growth factors can only be physically adsorbed, the binding forces of which are much smaller than chemisorption (Pan et al., 2019). When releasing, the growth factors physically absorbed on the titanium sheet and graphene release easily. After 14 days, the growth factors on the titanium plates were almost released, while still a lot of growth factors remained on the surface of the graphene-coated titanium plates, especially **S1** and **S2**.

### Morphological Changes and Osteogenic Differentiation of BMSCs on Graphene-Coated Titanium Sheets

**Figure 5:**
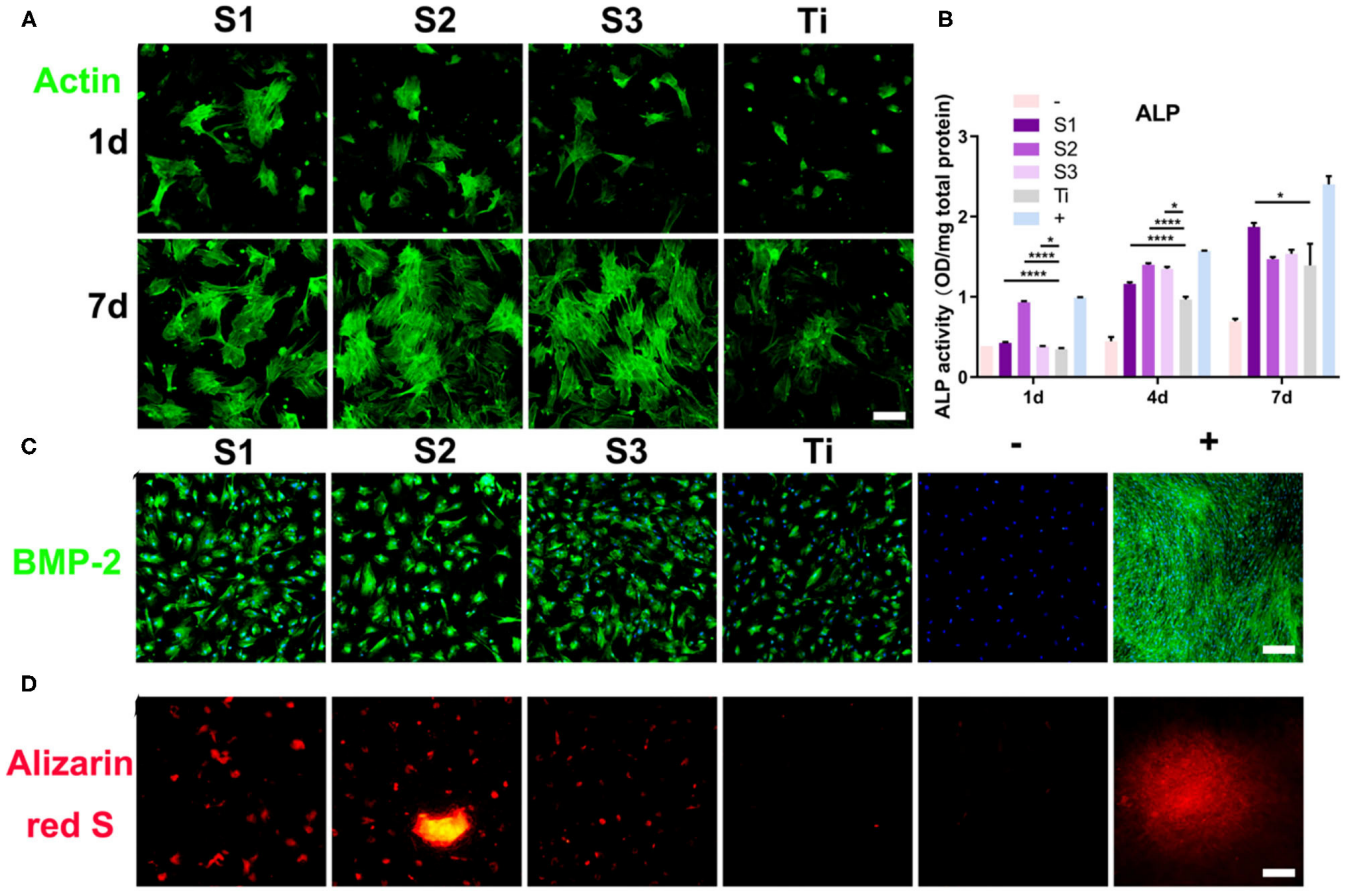
Morphological changes and osteogenic differentiation of BMSCs on the graphene-coated titanium sheets. **(A)** Actin filament network of cell morphologies on graphene-coated titanium sheets and titanium sheet, 24 h and 7d after seeding. **(B)** ALP activity of BMSCs at 1, 4, and 7 days on different samples. **(C)** Immunofluorescence staining for BMP-2 in differentiated BMSCs at 15 days and **(D)** alizarin red S (ARS) staining for mineralized nodules at 21 days. –: BMSCs on glass slides with growth medium. Graphene-coated titanium sheets (**S1–S3**) and titanium sheet (**Ti**) were treated with growth medium. +: BMSCs on glass slides with osteogenic medium (*p < 0.05 and ****p < 0.0001).

On the surface of graphene-coated titanium sheets, there were more cellular micro extensions and larger extension areas, and actin filaments had regular directions (Figure 5A). This indicates that BMSCs have better adaptability, proliferation and differentiation ability on the surface of the material, which may be due to the higher specific surface area, wrinkled surface morphology, mechanical property, electrical conductivity, reasonable number of functional groups, and more growth factors adsorbed on the graphene-coated titanium sheets.

Alkaline phosphatase (ALP) regulates the metabolism of organic or inorganic phosphates through the hydrolysis of phosphate esters. As a plasma membrane transporter of inorganic phosphates, ALP can be used as an early marker of osteoblast differentiation. In this study, the ALP activity of the cells cultured on graphene-coated titanium sheets increased significantly on the first day after incubation, which was close to that of the osteogenic medium group (Figure 5B). At 4 and 7 days after incubation, ALP level of cells on graphene-coated titanium sheets was also higher than that of control titanium sheet group.

Immunofluorescence staining of BMP-2 also confirmed the enhancement of osteogenic differentiation. Figure 5C shows that the immunofluorescence expression of the proteins increased significantly on the 15th day after incubation on the graphene-coated titanium sheets. The cells on the control glass slide showed weak fluorescence, while the expression on the titanium plate was also relatively weak. These results indicate that the adsorption of concentrated growth factors on graphene-coated titanium sheets is enough to induce the osteogenic differentiation of BMSCs. The positive Alizarin Red S staining strongly supports our findings that graphene-coated titanium sheets can effectively promote the osteogenic differentiation and calcium deposition of stem cells (Figure 5D), and enhance the early osteoblast differentiation.

### Gene Expression During Differentiation of BMSCs on Graphene-Coated Titanium Sheets

**Figure 6:**
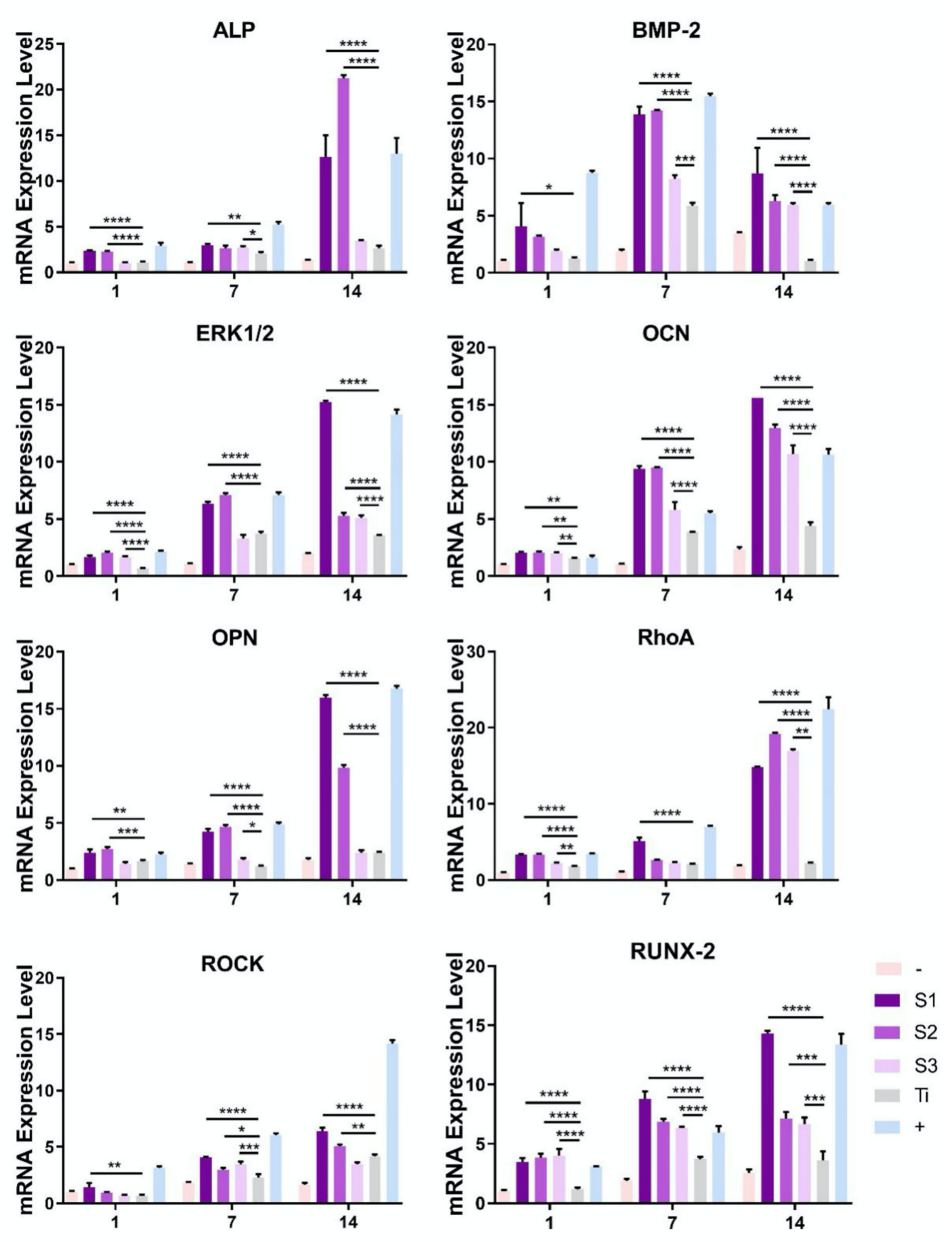
Quantitative RT-PCR analyses for the expression of genes mediating osteogenesis and differentiation processes. –: BMSCs on glass slide with growth medium. Graphene-coated titanium sheets (**S1–S3**) and titanium sheet (**Ti**) were treated with growth medium. +: BMSCs on glass slide with osteogenic medium. Scale bars: 50 μm (*p < 0.05, **p < 0.01, ***p < 0.001, and ****p < 0.0001).

Specific osteoblast mRNA expression was used to evaluate the differentiation of BMSCs into osteoblast lineage (Figure 6). The negative control of each experiment was cells cultured on glass slides (control group), and no osteogenic specific genes were detected. In addition, osteogenic medium was used as positive control to induce osteogenic differentiation on glass slides, resulting in significantly higher osteogenic specific gene expression. We first detected the expression of ALP, a marker of early osteoblast differentiation. From the first day, ALP expression level of BMSCs on the graphene-coated titanium sheets (**S1** and **S2**) was much higher than that of titanium sheet group, and the expression was similar on day 7 and 14. BMP-2 is an early marker of osteogenic differentiation. We found that the expression of BMP-2 in the cells seeded on graphene-coated titanium sheets was significantly higher than that on titanium sheet on day 7 and 14. The expression of ERK1/2, OCN, OPN, RhoA, ROCK, and RUNX-2 showed the same trend.

## Discussion

We used SLA coarsening to form a porous structure of about 2 μm on the titanium sheet. After modified by APTES solution, rGO sheets with diameter of 300–500 nm were loaded on the titanium surface, covalent bonds were formed between rGO and APTES, which could promote the rGO sheet to adhere to the porous structure as much as possible, and ultrasonic vibration could not peel them off. On the three concentrations of graphene-coated titanium sheets (0.4, 0.04, and 0.004 mg/ml), the cells showed spreading and confluence phenomenon, which indicated that the materials had good biocompatibility.

Graphene has good osteogenic ability, which is largely attributed to its surface morphology and mechanical properties (Borghi et al., 2018). Surface morphology of materials is the decisive factor of osteogenic differentiation. It has been reported that rough and disordered surfaces can induce osteogenic differentiation (Wall et al., 2009; Sun et al., 2018). The corrugated and porous surface of graphene can also provide anchor points for cytoskeleton, and affect the cytoskeleton tension, thus changing the cell morphology. It has been reported that cell morphology regulates the transition of lineage commitment by regulating endogenous RhoA, which is an important small G protein involved in cell signal transduction and cytoskeleton (McBeath et al., 2004). The results showed that the RhoA/ROCK1/ERK1/2 signaling pathway related to cytoskeleton deformation was also up-regulated. The cells with flat and well-diffused shape will undergo osteogenesis, and BMSCs on graphene-coated titanium sheets will also osteogenic differentiate.

With the increase of rGO coating concentration, the oxygen-containing groups such as hydroxyl groups, epoxy compound and carbonyl groups, as well as the conductivity increase. Graphene coating can optimize the adsorption capacity of materials by improving static electricity and van der Waals force, interact with autologous concentrated growth factors, and successfully adsorb and continuously release a variety of growth factors related to osteogenesis. There are more growth factors remaining on the surface of the materials, which can significantly promote the expression of ALP, BMP-2, RUNX-2, OCN, and OPN of the stem cells on the surface of the materials, suggesting that the materials can promote bone formation earlier and more strongly. Optimum graphene concentration ensures the appropriate amount of growth factors will be adsorbed and release to cooperate with the surface morphology to promote osteogenic. For cell morphology, ALP activity, expression of genes and the amount of growth factors adsorbed and released, we considered the concentration of S2 (0.04 mg/ml) to be the appropriate option.

In summary, rGO was successfully coated on SLA treated titanium surfaces, and graphene-coated titanium sheet surfaces showed good biocompatibility and distinguished capability of adsorbing and sustained releasing several osteogenic related growth factors. BMSCs on the graphene-coated titanium sheet were flattened and expressed more obvious osteogenic related genes and proteins compared to pure titanium, owing to the fact that graphene coating mediated cytoskeletal deformation through the RhoA/ROCK1/ERK1/2 signaling pathway to accelerate the osteogenic differentiation of BMSCs. In addition, the adsorption of various growth factors on the surface of graphene-coated titanium sheet could further promote the osteogenic differentiation ability of BMSCs. The rGO coating with concentrated growth factors was shown to facilitate osteogenic differentiation of implants and to be a potential substance for the modification of dental implants and a scaffold for bone regeneration.

## Data Availability Statement

The raw data supporting the conclusions of this article will be made available by the authors, without undue reservation.

## Ethics Statement

The animal study was reviewed and approved by Animal Welfare Ethics Committee of Shanghai Sixth People's Hospital.

## Author Contributions

SY and JL conceived the original idea and led the project. JL, JS, and DZ performed most of the assays. JL, JS, and SY performed data analysis and wrote the manuscript. All the authors have approved it for publication.

## Conflict of Interest

The authors declare that the research was conducted in the absence of any commercial or financial relationships that could be construed as a potential conflict of interest.

## References

[B1] Borghi F. F. Bean P. A. Evans M. D. M. van der Laan T. Kumar S. Ostrikov K. (2018). Nanostructured graphene surfaces promote different stages of bone cell differentiation. *Nanomicro*. Lett. 10:47. 10.1007/s40820-018-0198-0 30393696 PMC6199093

[B2] Chen F. M. Zhang M. Wu Z. F. (2010). Toward delivery of multiple growth factors in tissue engineering. Biomaterials 31, 6279–6308. 10.1016/j.biomaterials.2010.04.053 20493521

[B3] Chrcanovic B. R. Albrektsson T. Wennerberg A. (2014). Reasons for failures of oral implants. J. Oral Rehabil. 41, 443–476. 10.1111/joor.12157 24612346

[B4] Dubey N. Bentini R. Islam I. Cao T. Castro Neto A. H.. (2015). Graphene: a versatile carbon-based material for bone tissue engineering. Stem Cells Int. 2015:804213. 10.1155/2015/804213 26124843 PMC4466492

[B5] Holt B. D. Wright Z. M. Arnold A. M. Sydlik S. A. (2017). Graphene oxide as a scaffold for bone regeneration. Wiley Interdiscip. Rev. Nanomed. Nanobiotechnol. 9:e1437. 10.1002/wnan.1437 27781398

[B6] Li D. Ferguson S. J. Beutler T. Cochran D. L. Sittig C. Hirt H. P. (2002). Biomechanical comparison of the sandblasted and acid-etched and the machined and acid-etched titanium surface for dental implants. J. Biomed. Mater. Res. 60, 325–332. 10.1002/jbm.10063 11857440

[B7] Liao C. Li Y. Tjong S. C. (2018). Graphene nanomaterials: synthesis, biocompatibility, and cytotoxicity. Int. J. Mol. Sci. 19:3564. 10.3390/ijms19113564 30424535 PMC6274822

[B8] Lu J. Cheng C. He Y. S. Lyu C. Wang Y. Yu J.. (2016). Multilayered graphene hydrogel membranes for guided bone regeneration. Adv. Mater 28, 4025–4031. 10.1002/adma.201505375 27031209

[B9] McBeath R. Pirone D. M. Nelson C. M. Bhadriraju K. Chen C. S. (2004). Cell shape, cytoskeletal tension, and RhoA regulate stem cell lineage commitment. Dev. Cell 6, 483–495. 10.1016/S1534-5807(04)00075-9 15068789

[B10] Mourao C. F. Valiense H. Melo E. R. Mourao N. B. Maia M. D. (2015). Obtention of injectable platelets rich-fibrin (i-PRF) and its polymerization with bone graft: technical note. Rev. Col. Bras. Cir. 42, 421–423. 10.1590/0100-69912015006013 26814997

[B11] Murray P. E. (2018). Platelet-rich plasma and platelet-rich fibrin can induce apical closure more frequently than blood-clot revascularization for the regeneration of immature permanent teeth: a meta-analysis of clinical efficacy. Front. Bioeng. Biotech. 6:139. 10.3389/fbioe.2018.00139 30364277 PMC6193104

[B12] Pan S. Qi Z. Li Q. Ma Y. Fu C. Zheng S.. (2019). Graphene oxide-PLGA hybrid nanofibres for the local delivery of IGF-1 and BDNF in spinal cord repair. Artif. Cell Nanomed. Biotechnol. 47, 651–664. 10.1080/21691401.2019.1575843 30829545

[B13] Ryan A. J. Kearney C. J. Shen N. Khan U. Kelly A. G. Probst C.. (2018). Electroconductive biohybrid collagen/pristine graphene composite biomaterials with enhanced biological activity. Adv. Mater 30:e1706442. 10.1002/adma.201706442 29504165

[B14] Simonpieri A. Del Corso M. Vervelle A. Jimbo R. Inchingolo F. Sammartino G.. (2012). Current knowledge and perspectives for the use of Platelet-Rich Plasma (PRP) and Platelet-Rich Fibrin (PRF) in oral and maxillofacial surgery part 2: bone graft, implant and reconstructive surgery. Curr. Pharm. Biotechnol. 13, 1231–1256. 10.2174/138920112800624472 21740370

[B15] Stephens-Fripp B. Sencadas V. Mutlu R. Alici G. (2018). Reusable flexible concentric electrodes coated with a conductive graphene ink for electrotactile stimulation. Front. Bioeng. Biotech. 6:179. 10.3389/fbioe.2018.00179 30560123 PMC6286993

[B16] Su J. Du Z. Xiao L. Wei F. Yang Y. Li M.. (2020). Graphene oxide coated titanium surfaces with osteoimmunomodulatory role to enhance osteogenesis. Mater. Sci. Eng. C Mater. Biol. Appl. 113:110983. 10.1016/j.msec.2020.110983 32487397

[B17] Sun M. Chi G. Li P. Lv S. Xu J. Xu Z.. (2018). Effects of matrix stiffness on the morphology, adhesion, proliferation and osteogenic differentiation of mesenchymal stem cells. Int. J. Med. Sci. 15, 257–268. 10.7150/ijms.21620 29483817 PMC5820855

[B18] Tayalia P. Mooney D. J. (2009). Controlled growth factor delivery for tissue engineering. Adv. Mater. 21, 3269–3285. 10.1002/adma.200900241 20882497

[B19] Varkey M. Gittens S. A. Uludag H. (2004). Growth factor delivery for bone tissue repair: an update. Expert. Opin. Drug Deliv. 1, 19–36. 10.1517/17425247.1.1.19 16296718

[B20] Wall I. Donos N. Carlqvist K. Jones F. Brett P. (2009). Modified titanium surfaces promote accelerated osteogenic differentiation of mesenchymal stromal cells *in vitro*. Bone 45, 17–26. 10.1016/j.bone.2009.03.662 19332166

[B21] Wu Z. Wang Y. Liu X. Lv C. Li Y. Wei D.. (2019). Carbon-nanomaterial-based flexible batteries for wearable electronics. Adv. Mater. 31:e1800716. 10.1002/adma.201800716 30680813

[B22] Xing J. Tao P. Wu Z. Xing C. Liao X. Nie S. (2019). Nanocellulose-graphene composites: a promising nanomaterial for flexible supercapacitors. Carbohyd. Polym. 207, 447–459. 10.1016/j.carbpol.2018.12.010 30600028

[B23] Yan S. Zhu X. Frandsen L. H. Xiao S. Mortensen N. A. Dong J.. (2017). Slow-light-enhanced energy efficiency for graphene microheaters on silicon photonic crystal waveguides. Nat. Commun. 8:14411. 10.1038/ncomms14411 28181531 PMC5309776

